# The effectiveness of an augmented cognitive behavioural intervention for post-stroke depression with or without anxiety (PSDA): the Restore4Stroke-PSDA trial

**DOI:** 10.1186/1471-2377-12-51

**Published:** 2012-07-07

**Authors:** Joyce A Kootker, Luciano Fasotti, Sascha MC Rasquin, Caroline M van Heugten, Alexander CH Geurts

**Affiliations:** 1 Department of Rehabilitation, Nijmegen Centre for Evidence Based Practice, Radboud University Nijmegen Medical Centre, Nijmegen, The Netherlands; 2 Donders Institute for Brain, Cognition and Behaviour, Radboud University Nijmegen, Nijmegen, The Netherlands; 3 Rehabilitation Medical Centre Groot Klimmendaal, Arnhem, The Netherlands; 4 Adelante Rehabilitation Foundation Limburg, Limburg, The Netherlands; 5 Dept Rehabilitation, CAPHRI, Maastricht University Medical Centre, Maastricht, The Netherlands; 6 Department of Psychiatry and Neuropsychology, School for Mental Health and Neuroscience, Faculty of Health, Medicine and Life Sciences, Maastricht, The Netherlands; 7 Department of Neuropsychology and Psychopharmacology, Faculty of Psychology and Neuroscience, Maastricht University, Maastricht, The Netherlands

**Keywords:** Post-Stroke Depression, Cognitive Behavioural Therapy, Anxiety

## Abstract

**Background:**

Post-Stroke Depression with or without Anxiety (PSDA) is a common disorder in the chronic phase of stroke. Neuropsychiatric problems, such as PSDA, have a negative impact on social reintegration and quality of life. Currently, there is no evidence-based treatment available for reducing PSDA symptoms. In the recent literature on depression in the general population it has been shown that depression complaints can diminish by cognitive behavioural therapy (CBT). In the current study, the effectiveness of augmented, activation-based and individually tailored CBT on the reduction of depression and anxiety will be investigated in patients with PSDA. Additionally, the effects on various secondary outcome measures, such as quality of life, goal attainment and societal participation will be evaluated. This study is embedded in a consortium of 4 interrelated studies on quality of life after stroke (Restore4Stroke).

**Methods/design:**

A multi-centre, assessor-blind, randomized controlled trial is conducted. A sample of 106 PSDA patients, as assessed with the Hospital Anxiety and Depression Scale (HADS depression subscale >7), will be recruited and randomly allocated to either an experimental or a control group. The experimental intervention consists of an augmented CBT intervention. The intervention is based on CBT principles of recognizing, registering, and altering negative thoughts and cognitions so that mood, and emotional symptoms are improved. CBT is augmented with direct in-vivo activation offered by occupational or movement therapists. Patients in the control group will receive a computerized cognitive training intervention. Outcomes will be assessed at baseline, immediately post intervention, and at 6 and 12 months follow up.

**Discussion:**

This study is the first randomized clinical trial that evaluates the (maintenance of) effects of augmented CBT on post-stroke depression with or without anxiety symptoms. Together with three other projects, the Restore4Stroke PSDA trial will provide novel information about the (treatment of) emotional problems and quality of life after stroke.

**Trial registration:**

Trial registration number: Dutch Trial Register NTR2999

## Background

Stroke causes a high burden of disease to patients as well as to their caregivers. One year after a stroke, 35% of the patients are functionally dependent, indicating that stroke is a leading cause of disability [1]. Half of all patients has problems in returning to their pre-stroke daily routines [2]. Particularly the ensuing psychosocial problems of stroke survivors have a negative impact on their quality of life [3]. Emotional adjustments are associated with changes in coping strategies which are related to quality of life [4,5]. Additionally, it has been shown that caregivers experience substantial burden from their partners’ emotional problems as well [6]. Cognitive and emotional complaints are found in many patients in the chronic phase after stroke [7]. Within the first three months, post-stroke depressive symptoms can spontaneously resolve. If this does not occur, these symptoms can take a chronic course [8]. In the chronic phase (usually defined as > 6 months post stroke), the most frequent emotional symptoms are depression (23-25%) and anxiety (19-23%) [9-12]. Post-stroke depression and anxiety often co-occur and interact, but may also be present in isolation. Apparently the post-stroke prevalence of anxiety is almost as high as it is for depression; hence, it is surprising that the literature largely focuses on post-stroke depression (PSD) [9-12]. Stressing the impact and co-occurrence of anxiety on patients with stroke, in the present study, we introduce the new concept of ‘Post-Stroke Depression with or without Anxiety’ (PSDA).

Currently, we are not aware of any treatment studies on post stroke anxiety. Studies on the treatment of PSD have generally focused on pharmacological interventions [13,14], whereas psychological interventions for PSD have hardly been investigated [15]. Several researchers have investigated the possibility of preventing depression after stroke [16-19]. Different interventions were evaluated, such as meditation, breathing exercises and visualisation (i.e., encouraging a new way of thinking about disability) [16] and visits by specialist outreach nurses providing information, advice and support [17]. In the intervention studies, a small but statistically significant reduction in psychological distress was reported on a variety of health-related questionnaires. All intervention studies were based on contact with multiple disciplines providing information and support, yet no specific psychological therapies were administered.

Nevertheless, there are indications that the treatment of depressive symptoms using cognitive behavioural therapy (CBT) is effective in other chronic illnesses such as cancer and diabetes [20,21]. Hence, psychological interventions seem promising in terms of effectiveness, while they have fewer side effects than medication. Moreover, as CBT is aimed at changing irrational cognitions and negative thoughts, it is considered to have a stronger effect on preventing relapse of symptoms than pharmacotherapy [5,16,20,22,23]. In one of the studies that dealt with treatment of PSD, Lincoln and colleagues, applied a treatment protocol in which identifying and challenging negative thoughts as well as planning of joyful activities was discussed with the patients. The authors found, however, inconclusive results with respect to the effectiveness of such a psychological intervention [24,25]. CBT requires effort during treatment sessions and often comes with a substantial amount of homework. The inconclusive results could be due to the inclusion criteria that did not take into account cognitive deficits and impaired awareness after stroke. These factors are associated with poorer rehabilitation outcomes [26] and are, therefore, deemed essential when applying CBT. Next to discussing activation with the patients, no further steps, such as stimulating an active life style or giving practical suggestions and encouragements directed at joyful activities, were taken by Lincoln and colleagues [25]. Including such an extra step in an intervention could possibly catalyse generalisation of the content of the therapy to daily-life functioning [15]. Furthermore, in a study with brain-injured patients, a combination of CBT and individual cognitive remediation seemed to diminish psychological distress (e.g., depression, anxiety, coping) and improve attention and cognitive functions. Nevertheless, which of the two therapy ingredients was (most) effective could not be determined [27]. Implementation of CBT should take into account cognitive impairments as well as emotional barriers and awareness. To stimulate treatment generalisation, the use of behavioural exercises that have to be executed in daily life situations is recommended [15].

Taken together, the scientific literature shows inconsistent results on the effectiveness of treatment programs for PSD. Despite a lack of clear evidence, there is sufficient reason to believe that CBT offered by a trained psychologist can be effective in PSDA when adapted and tailored to the specific needs of patients with stroke, as discussed above [15]. Combining the results of earlier research on treatment and prevention of post-stroke depression and according to the recommendations expressed in these studies [7], an augmented psychological approach to CBT will be tested in the present study using a Randomised Controlled Trial (RCT) design. An equally intensive control intervention (i.e., computerized cognitive training) was selected with the prospect of improving intervention-related goals (e.g., attention, memory, executive functioning) and attaining previously reported high patient satisfaction [28].

The present study is a multi-centre randomized controlled trial (RCT). The primary objective is to evaluate the direct and long-term intervention effects on PSDA symptoms as assessed with the Hospital Anxiety and Depression Scale (HADS). It is hypothesised that patients treated with augmented CBT, tailored to their needs, will show a larger decrease in HADS scores than those receiving computerized cognitive training. The secondary objectives are:

to evaluate the effects on societal participation, quality of life, goal attainment and coping strategies. It is hypothesised that the augmented CBT will lead to better outcomes in all these domains.

to assess the level of burden of the patients’ caregivers. It is expected that the augmented CBT will be more beneficial with respect to alleviating the perceived burden (emotional as well as practical) of caregivers.

to evaluate the relationship between treatment participation of the patients in CBT on the one hand and treatment outcomes on the other hand.

The present study is part of a comprehensive rehabilitation research program, Restore4Stroke, in the Netherlands (http://www.restore4stroke.nl) comprising four interrelated studies. Restore4stroke aims to monitor, predict and improve the quality of life of patients and their caregivers. Besides the present RCT, three other studies are conducted: a longitudinal cohort study [29], an RCT aimed at evaluating the effect of a self-management group therapy, and an economical evaluation study [30]. The design of the other studies will be published separately.

## Methods

### Study population

We intend to include 106 participants who experience depressed mood with or without symptoms of anxiety after stroke. When physicians, therapists or psychologists in the Netherlands notice such complaints during the rehabilitation process or thereafter, patients can be referred to various rehabilitation centres offering the experimental and control interventions distributed throughout the country. Participants may also be referred by other healthcare professionals or come on their own initiative based on ‘hear say’. They will be eligible if they have sustained any type of stroke in the past, if complaints developed after the stroke and if their depression sub-score on the Hospital Anxiety and Depression Scale (HADS) is above seven. Other inclusion criteria are: (1) being more than three months post stroke; (2) being 18 years or older; (3) having sufficient communication skills (MMSE > 27, communication-related NIHSS items); and (4) mastering the Dutch language. Exclusion criteria are: (1) pre-morbid disability as assessed with the Barthel Index (score < 19/20); (2) stay in an inpatient setting; (3) co-morbidity that might affect outcome (e.g., cancer or major psychiatric illnesses for which psychological treatment is given at the moment of inclusion); (4) a major depression diagnosis requiring medication; and (5) a pre-morbid major depression diagnosis, or having received psychiatric care for depression.

### Procedure

Participants will be screened, enrolled and subsequently treated in several rehabilitation centres or rehabilitation departments of hospitals in the Netherlands. They will be recruited over a period of one year. Each centre is expected to enrol at least 10 patients. The physicians of the affiliated centres are provided with information about the inclusion and exclusion criteria to perform a first test of eligibility (HADS assessment). When individuals are considered to be candidates for the study, they will be informed about the Restore4Stroke PSDA trial by written correspondence and will be asked for permission to be contacted by the primary investigator (JK). In the case of a positive reply, the primary investigator will make an individual appointment to confirm the inclusion and exclusion criteria, to obtain oral and written informed consent, and to perform the baseline assessment (T0).

**Figure 1:**
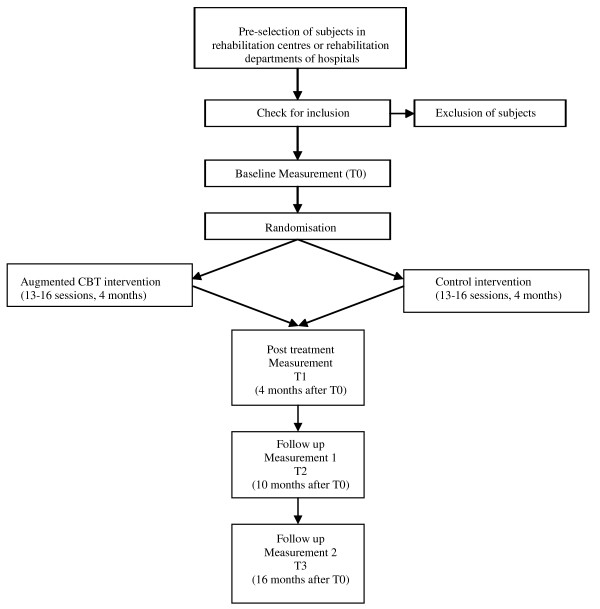
Flow chart of trial design.

Participants will be randomly allocated to an experimental group receiving augmented CBT or to a control group receiving computerized cognitive training. All outcome measures will be collected at baseline (T0), after 4 months (post treatment) (T1), and at 6 (T3) and 12 months (T4) follow up (see Figure 1). All assessments will take place in the (patients’ nearest) participating centres. From T1 assessments will be performed by research assistants who are not involved in the delivery of the interventions and who will be blind to the type of intervention in individual participants. Prior to each assessment, the assessor will explicitly ask the participant not to reveal or discuss the content of his/her intervention. Successfulness of assessor blinding will be tested by the primary investigator at the end of each individual intervention period using a short self-constructed questionnaire. During the intervention it is required that any antidepressant medication intake remains stable. Yet, when changes in medication occur these will be registered. This procedure was approved by the Committee on research involving Human Subjects, Nijmegen, The Netherlands, and all local participating centres.

### Randomization

Stratified block randomization (block size n = 4) will take place by a computer in each participating rehabilitation centre separately. Participants will be stratified according to their anxiety level (cut-off: HADS subscale anxiety score ≤7 vs. > 7). In this way, all blocks will assumedly consist of an equal number of participants with and without anxiety complaints and each participating centre will provide both interventions in approximately an equal number of participants.

### Interventions

#### Augmented cognitive behavioural therapy

Subjects in the experimental group will be treated with an augmented CBT protocol using ‘CRASS’ communication techniques [31]. The ‘CRASS’ techniques entail Concrete, Repetitive, Accessible, Structured and Specific communicative principles. Therapists will use motivational interviewing and interpersonal therapy techniques. Patients will be taught to recognize, register, and alter negative thoughts, cognitions and concurrent feelings. To encourage daily life integration of the CBT, content sheets will be provided and related homework will be given each session (i.e., one or two assignments per session). The intervention is individually administered and tailored according to specific subjects-own activity-related goals. The treatment program consists of 10–12 sessions with an experienced health-care psychologist and is augmented with three (or four; depending on HADS anxiety scores) sessions of occupational therapy (OT) or movement therapy (MT). These latter sessions will be provided concurrently with the psychological sessions and serve as a facilitator for reaching the pre-set goals in daily life. Therapy goals will be set in terms of increased participation in meaningful or joyful activities using Activity Card Sorting (ACS) [32]. This tool is incorporated to overcome problems with goal-setting because of the passive state PSDA patients can be in. With the ACS, patients can objectify their goals with the help of pictures/photos that represent elderly participating in various activities. In this way, patients are given concrete and accessible incentives to define their own personal goals more easily. The OT/MT will help to initiate and guide patients to execute goal-related activities. If patients’ HADS anxiety sub-score is >7, the protocol will be expanded with an additional (fourth) OT/MT session. Therapists will be informed about the HADS anxiety sub-scores of the patient. The additional session entails relaxation techniques as provided by the Dutch Heart Foundation. The OT/MT can refer to the relaxation techniques if patients report experienced anxiety during execution of their activities [33]. Next to that, psychologists will address anxiety-related thoughts, cognitions and concurrent feelings when executing CBT.

The protocol framework is set out in five successive phases (see Table 1). The therapist will start with building rapport creating a safe atmosphere. Psycho-education about current mood states will guide and prepare patients for the next phase: Goal setting. Subsequently, CBT will be executed using explanatory schemes about negative and positive cognitions [34]. Themes of the CBT range within patients-own goals and concurrent mood states. From this stage the OT/MT will actively work with patients on their treatment goals as well. CBT succeeds with continuation of learned techniques and concludes with building a relapse plan for future challenging situations.

**Table 1:** Content of successive phases of the augmented Cognitive Behavioural Therapy

**Phase**	**Content**
1. Introduction	Building rapport; discussing grief; psycho-education about the relationship between mood and behaviour.
2. Goal setting	Active goal setting with OT/MT.
3. CBT principles	Recognising and registration of ‘hidden cognitions’.
4. CBT	Challenging and altering ‘negative cognitions’.
5. Relapse prevention	Building a relapse-prevention plan with taught techniques.

All treatment sessions are supervised by certified health-care psychologists who have sufficient experience with treatment of depression as well as with stroke rehabilitation. The psychologist will supervise the OT/MT and will determine the sequence of the treatment sessions according to the individual needs and capacities of the subjects. The program is executed within a four-month time span, with a minimum of 13 and a maximum of 16 sessions. Each session will take two 20–25 minute-blocks divided by a 10–15 minute break. Treatment will be given at the most nearby participating centre.

#### CogniPlus; computerized cognitive training

Subjects in the control group will be given a computerized cognitive training program (CogniPlus). The amount of training sessions will be equal to that of the experimental group (i.e., 13–16 sessions). The control intervention is individual and patient-tailored as well. Participating centres will be provided with software and a dongle with sufficient training hours. With headphones and coloured patches attached to two keys on the keyboard, a desktop can be set up for CogniPlus execution. Patients register on the system and for each session, content and results of the training will be saved. The program is an impairment-oriented intervention which suits the stroke population. In specific self-determined cognitive domains (i.e., attention, memory, executive functioning, and visual attention) patients will be executing computer tasks on performance-own related levels. A research or psychological assistant will be present during the training sessions for support or to answer any questions about the intervention. This person will, however, not engage in conversations with the patients about topics other than the cognitive training. To assess deterioration of mood, Visual Analogue Scales (VAS) will be filled out by the participant after each session. The assistant will monitor VAS scores and, when substantial deterioration occurs, will notify the researcher and the psychologist of the department.

### Outcome assessment

#### Patients

Primary and secondary outcome measures are listed in Table 2. The HADS is a depression and anxiety questionnaire developed for use in patients with somatic co-morbidity such as stroke. We will assess domain-specific as well as health-related quality of life. The primary outcome measure is the depression subscale of the HADS [35,36]. Health-related quality of life will be assessed in general (EQ6D) [37], and stroke specific (SSQoL) [38]. Domain-specific quality of life is represented by participation assessment (USER-P) [39], Emotional functioning (HADS), and subjective well-being (life satisfaction questionnaires).

**Table 2:** Outcome measures

	**Tests**
**Primary outcome measure**	
Depression and Anxiety	Hospital Anxiety and Depression Scale (HADS)
**Secondary outcome measures**	
Quality of Life	
General	Stroke Specific quality of Life (SSQoL-12)
	EuroQuality of Life (EQ6D)
Domain specific	User-P
	Life Satisfaction questions
Post-stroke depression	Post Stroke Depression Rating Scale (PSDRS)
Goal attainment	Goal Attainment Scale (GAS)
Participation in therapy	Pittsburgh Rehabilitation Participation Scale (PRPS)
Coping strategy	Utrecht Proactive Coping Competence scale (UPCC)
**Caregiver outcome measures**	
Domain specific quality of life	Hospital Anxiety and Depression Scale (HADS)
Subjective strain	Caregiver Strain Index (CSI)
Emotional burden	Involvement Evaluation Questionnaire (IEQ)

Next to these quality of life related assessments, a semi-structured questionnaire specifically regarding post-stroke depression will be used. The Post Stroke Depression Rating Scale (PSDRS) will accompany HADS results emphasizing the phenomenological aspects of post-stroke depression [40]. This scale comprises a characterization of all depressive post stroke symptoms (e.g., psychological, physical and vegetative aspects). CBT is aimed at different aspects of coping such as regulating emotional reactions and reassuring thoughts (emotion-focused coping), changing stressful situation and learning new skills (problem-focused coping) [41]. Consequently, we will assess coping using the Utrecht Proactive Coping Competence scale (UPCC) [42]. Goal setting will be performed with the therapist, and Goal Attainment Scaling (GAS) will be carried out by the primary investigator (JK) [43]. Treatment participation will be scored by the therapist per session. The Pittsburgh Rehabilitation Participation Scale (PRPS) is a short quantitative measure that is easy to administer [26]. The PRPS will be completed in each session and can provide: 1) direct feedback on individual treatment participation for the therapist, 2) direct feedback on treatment participation per session in general. If we come across trends in reported participation, the protocol could be adjusted.

#### Caregivers

In addition to patient-related outcomes we will also assess caregivers’ emotional and practical burden with the HADS, the Involvement Evaluation Questionnaire (IEQ) [44] and the Caregiver Strain Index (CSI) [6,45,46].

### Statistical analysis

Statistical analysis will be performed using PASW 18. Baseline characteristics will be presented using descriptive statistics. Differences between groups at baseline will be tested using chi-square and t-tests, depending on the type of variable. General linear model (GLM) analysis will be used to investigate the main effects of Time and Group (experimental vs. control) and the Time x Group interaction. Because it is hypothesized that augmented CBT causes a greater effect on the HADS than cognitive training and that this effect will last at least 6 months after cessation of the therapy, the primary endpoint will be at 6 months post treatment (T2). Data analysis will be performed by an independent statistician who will be blinded for group intervention. Data will be analysed according the intention-to-treat principle. Alpha will be set at 0.05.

### Power

With a minimally detectable effect size of 0.6 SD on the HADS, α = .05 and β = .80, minimally 45 participants in each group are required. With an expected drop-out rate of 15%, 106 participants will need to be recruited.

## Discussion

Currently, no multi-centre RCT has been conducted that has evaluated the effectiveness of an (augmented) CBT intervention on post-stroke depression with or without anxiety [47]. Reduction of PSDA could positively influence psychosocial functioning, rehabilitation outcome and concurrently improve different domains of quality of life. An innovative aspect of the current study is that the CBT intervention takes into account recommendations by Broomfield and colleagues for the design of a post-stroke CBT intervention [15]. Specifically, the proposed CBT is augmented with occupational therapy or movement therapy to facilitate change of behaviour in real life by promoting and encouraging meaningful or pleasurable activities. This augmentation may, however, be a challenging factor in some individuals with lack of initiative. These individuals may find it difficult to cope with the amount of homework. Striving to overcome this problem, only therapists with sufficient experience with the stroke population will deliver the intervention, and treatment participation will be monitored. Next to that, the protocol continually emphasizes CRASS communication principles, and the ACS is offered as a tool to provide input for conversation about joyful activities. Another innovation in the present study is that depressive symptoms are used for inclusion of subjects, but that symptoms of anxiety are not considered a reason for exclusion. In contrast, anxiety complaints will be incorporated as a target for the proposed cognitive behavioural therapy.

An important methodological aspect of the present study is that subjects from different parts of the Netherlands are included, which will enable us to control for possible cultural differences between subgroups. Another methodological strength is that the augmented CBT is compared to an ‘active’ intervention (and not to ‘usual care’) to control for Hawthorne effects. Lastly, a parallel economic evaluation study within the Restore4Stroke program will shed light on the cost-effectiveness of the proposed intervention [30].

Taken together, the Restore4Stroke PSDA trial is an essential part of the Restore4Stroke program that focuses on the effectiveness of an augmented and individually tailored CBT protocol for treating post-stroke depression with or without anxiety. The Restore4Stroke program will: provide longitudinal information about the course of quality of life of patients and caregivers and the influence of personal factors on quality of life, evaluate two interventions designed for stroke patients, and last but not least will evaluate the economical effectiveness of both trials.

### Current study status

The Restore4Stroke PSDA trial has started recruitment from the beginning of 2012.

## Abbreviations

CBT, Cognitive Behavioural Therapy; EQ6D, Euro Quality of Life; GAS, Goal Attainment Scale; HADS, Hospital Anxiety and Depression Scale; MT, Movement Therapist; OT, Occupational Therapist; PRPS, Pittsburgh Rehabilitation Participation Scale; PSD, Post-Stroke Depression; PSDA, Post-Stroke Depression with or without Anxiety; PSDRS, Post-Stroke Depression Rating Scale; SSQoL, Stroke Specific Quality of Life; UPCC, Utrecht Proactive Coping Competence scale; VAS, Visual Analogue Scale.

## Competing interests

The authors declare that they have no competing interests.

## Authors’ contributions

JK is the primary investigator and will execute the study and monitor both interventions in all patients. JK is responsible for data collection, data analysis and for drafting the first manuscript. LF, AG, SR and CvH are designers and supervisors of the study. All authors will participate in finalizing the manuscript. All authors read and approved the final manuscript.

## Pre-publication history

The pre-publication history for this paper can be accessed here:

http://www.biomedcentral.com/1471-2377/12/51/prepub
